# Effects of Freezing Time on Degradation of Durian (*Durio Zibethinus* Murr.) Fruit’s Attributes During the Frozen Storage

**DOI:** 10.21315/tlsr2023.34.1.2

**Published:** 2023-03-30

**Authors:** Hendra Adi Prasetia, Slamet Budiawan, Ade Syahputra, Retno Umiarsih, Rifena Pangastuweni, Mutia Riefka Fauzidanty, Idham Sakti Harahap, Dondy Anggono Setyabudi, Affandi, Mazdani Ulfah Daulay, Wawan Sutian

**Affiliations:** 1 Applied Research Institute of Agricultural Quarantine, Indonesia Agricultural Quarantine Agency, Ministry of Agriculture, Jl. Raya Kampung Utan-Setu, Ds. Mekarwangi, Kec. Cikarang Barat, Kab. Bekasi 17520 Jawa Barat, Indonesia; 2 Department of Plant Protection, Faculty of Agriculture, IPB University, Jl. Kamper Babakan Dramaga, Kab. Bogor 16680 Jawa Barat, Indonesia; 3 Center for Agroindustry, Research Organization for Agriculture and Food, National Research and Innovation Agency, Gedung 614, Kawasan Puspitek Serpong, Tanggerang Selatan, Banten, Indonesia; 4 Center for Horticulture and Plantation, Research Organization for Agriculture and Food, National Research and Innovation Agency, Cibinong Science Center, Jl. Raya Jakarta-Bogor, Cibinong, Kabupaten Bogor 16915, Jawa Barat, Indonesia

**Keywords:** Freezing, Core Temperature, Medium Distribution, Fruit’s Attributes, Customer’s Preference

## Abstract

Freeze-process has been applied in preserving many fresh horticultural commodities addressed to the medium-distancing distribution. In this study, effect of freezing process and storage time on durian’s attributes degradation was observed. 100 durian fruits were treated with two-level combinations of freezing process. The first level involves the freezing of the said fruit at −15°C for two different freezing times, that is 10 min (treatment A) and 20 min (treatment B). Followed by frozen-storage for −10°C for 0, 10, 20 and 30 days. At different interval time, the frozen samples were thawed at 4°C for 24h. Then, physical, chemical, and sensory parameters were periodically assessed. The result showed that treatment B provide a significantly better output than treatment A. This is proven through a lower weight loss, brighter and lighter yellow of the pulp, softer pulp, lower value of moisture content on the pulp, and a remained stable of succinate acid’s profile. Furthermore, based on the preference evaluation test, the fruits were accepted by respondents.

**Highlight**

- The core freeze-process at −15**°**C for 20 min followed by the freezing one at −10°C for 30 days has been proved to the best scheme in maintaining the physics-chemical fruit’s properties.
- Sensory preferences have also been acceptable, at least until one month storage.
- This technical scheme has contributed in lengthening the shelf of life of durian, particularly for distributing this commodity in medium distance trade.

## INTRODUCTION

Durian (*Durio zibethinus Murray*) is one of the tropical horticultural commodities that is gaining considerable attention worldwide. This fruit is categorized as the super-premium class due to its yellow pulp color, its sweetness taste, and its unique ripening flavor (Ketsa *et al*. 2020; Somsri & Vichitrananda 2007). Thailand, Malaysia and Indonesia have been recognised as global producers of these fruits throughout the world (Subhadrabhandu & Ketsa 2001; Wilaipon 2011). Due to the global customers’ expansion, the most promising markets consist of a group of segmented customers demanding several local cultivars of durian with their own unique characteristics relating to their performances have increased (Leontowicz *et al*. 2008)

Based on physiological alterations, these cultivars exhibit a range of different indications for performing to their optimal maturity (Ketsa *et al*. 2020; Thongkum *et al*. 2018; Tifani *et al*. 2018). For instance, colour changing of the husk (yellowish green), producing specific durian-flavour at the abscission layer, softening of the spines as well as rising of stalk elasticity has distinctly indicated for demonstrating the initial maturity gradually levelling-up during the postharvest period (Ketsa & Daengkanit 1998; Pascua & Cantila 1992; Tongdee *et al*. 1990; Wattanawichean *et al*. 2002). Unfortunately, since durian is a tropical fruit, its maturity has a limited shelf life. Therefore, seeing freezing storage is a physical preservation method of fruits, it has spurred a wide range of opportunities for extending fruit supply chains, such as supporting export-import trade distribution (Arancibia-Avila *et al*. 2008; Yahia 2011).

As a tool for fruit preservation, freeze-processing offers many benefits. Based on several studies, reduced water activity appears to hinder microbial growth as well as decay of the fruit as a result of slowed enzymatic reactions that are critical to maximizing the shelf life of several frozen foods (Alhamdan *et al*. 2016; Sanz *et al*. 1999; Ratti 2001; Sun & Li 2003). Due to a higher relative humidity, the rate of frost formation increased rapidly in the initial period. A blockage in air circulating on the surface causes the formation of frost on the surface, which is especially destructive to the stability of the air-flow rate cascading on the surface of the frozen material (Ameen *et al*. 1993; Fisk *et al*. 1985; O’Neal *et al*. 1989). Furthermore, the partial moisture pressure has dropped drastically, which in turn has caused the freezing operation to become more work-intensive due to the fan operation (Amer & Wang 2017; Padki *et al*. 1989; Yan *et al*. 2005). To normalise the situation, a regular defrosting process was selected as a way to reduce the pressure gap. Therefore, there will no longer be condensate formed at excessive rate. Which is an improvement in freezing capacity scheduled in a timeframe of measured allocation (Dong *et al*. 2011; Jhee *et al*. 2002; Kim & Lee 2011).

Once the fruit leaves taken out of the freezer, the food undergoes a thawing process. Thawing the fruit by providing moisture near its freezing point has preserved its structure (Assegehegn *et al*. 2019; Martinez-Navarrete *et al*. 2019; Novak & Jakubczyk 2020). Therefore, fruit’s discoloration and its versatile texture will be less noticeable (Khalloufi & Ratti 2003; Krzykowski *et al*. 2018; Silva-Espinoza *et al*. 2019) and more acceptable to consumers (Tagubase *et al*. 2016; Zhang, Zhang, *et al*. 2007; Zhang, Duan, *et al*. 2004). Inducing hot moisture’s supply as a prime-medium has gained more positive effect in maximizing the specific flavour produced by the aromatic’s enzyme contained in many particular fruits, such as strawberry (Modise 2008), mango (Chassagne-Berces *et al*. 2010), peach (Chassagne-Berces *et al*. 2009), and dates (Alhamdan *et al*. 2016). There are relatively few published studies addressing the exposure of frozen environmental storage on the whole durian as a potential tropical horticultural product.

The objective of the research was to determine the effects of freeze-process and frozen storage period towards the physical as well as the chemical attributes of said fruit. Along with the sensory preferences during the storage. The research could be implemented on estimating the self-life and far away distribution as well. As a result, these data would be vital as a scientific basis for expanding market access for Indonesian durians.

## MATERIALS AND METHODS

### Time, Site Locations and Durian Fruits

This research was conducted at a research facility in Bekasi Regency (city in Java Indonesia) known as the Applied Research Institute of Agricultural Quarantine (ARIAQ) from January until December 2020. A total of 100 local durian fruits were used in this study. The local cultivar namely *Langgang Kamang* was traditionally cultivated in a local orchard called the R.E Durian Farm which is located at Selareh Aia urban-village, Palembayan sub-district, Agam Regency, West Sumatera Province 26164, Indonesia, with a global-map coordinate at −0.1209792,100.1085487. The durian fruits used in this research had a range of physiological maturity ranging from 85%–90% under regular cultivation sessions and territorial conditions in late August 2020. It took 12 h to transfer these fruits to the Applied Research Institute via a vehicle that was equipped with an air ventilator. The purpose of said ventilator was to ensure that the temperature within the vehicle was kept between 25°C–28°C until these fruits reached their final destination.

### Freeze-Process and Its Freezing Storage

Initially, 10 whole fruits were hand-picked as the control for further assaying of their physics-chemical and organoleptic properties. While another three were chosen for monitoring purposes, such as its temperature. A thermometer logger (K Type SD Card PH99, China), is inserted at a depth of 20 cm–25 cm inside the fruit until it reaches the pulp. The temperature monitoring process was applied to all 13 fruits, including the three fruits which has been selected to observe the pulp’s temperature. Locating 40 cm from the blowing cold-air source, then the 10 fruits were inserted into a refrigerated consignment (Daikin LXE-10E, Japan) for chilling at 4°C for 24 h.

The next process was freezing. The purpose of said process is to achieve the targeted temperature, which is −15°C. During this process, a series of regular observations was carried out to monitor the relationship between the reached temperature observed on the thermometer-probes with the certain time-process. As previously discussed, if the defrost-cycle was naturally occurring, as indicated by a noisy and abnormal engine sound, then it would take several periods to normalize this cycle by using the off-cycle defrosting method (Amer & Wang 2017; 2021; Zheng *et al*. 2020). Soon after the process was normalised, marked by regular and normal-engine sounds, then further observations were resumed up to the targeted temperature finally achieved. After that, calculation of time-process was commenced, subsequently for 10 min (as treatment A) and 20 min (as treatment B).

When the entire freezing process was accomplished, then the freeze-storage temperature was adjusted into −10°C for 30 days. In each of the 10 day-interval of storage time, a series of physics-chemical and sensory examinations were conducted to both, 15 pieces of fruits treated with the A-level and the same quantity of other fruits treated with the B-one, previously transferred into a fruit showcase (LG-800, South Korea) at 4°C for the thawing process, the day before.

### Weight Loss

This parameter was periodically calculated based on a method described by Nunez and Emond (2007).

### Moisture Content

This analytical procedure and its calculation were formulated based on AOAC (2000).

### Colour

Colour attributes measured on the pulp’s surface were determined by using a colorimeter (Konica Minolta CR 13, Japan). There are three observed parameters, such as: L (lightness), a (+a = red; −a = green), and b (+b = yellow; −b = blue) (Voon *et al*. 2006) read on the screen. Chroma value and hue angle have subsequently been calculated as follows:


$$\begin{array}{l} \text{chroma}={\left({\text{a}}^{2}+{\text{b}}^{2}\right)}^{\left(0.5\right)}\\ {\text{hue}}^{\circ }={\text{tan}}^{-1}\;\left(\frac{\text{b}}{\text{a}}\right) \end{array}$$

### Total Soluble Solids

The content of total soluble solids was periodically measured using a pocket refractometer (Atago Co, Japan) based on a method developed by Booncherm and Siriphanich (1991).This observation was carried out on the flesh of the durian fruit. By squeezing the flesh until the fruit liquid came out, the three parameters were observed on the prism of a hand-refractometer. Observations were made at each interval of the storage period.

### Determining of Fruit Firmness

A whole fruit was peeled. Then, the fleshes of fruit were released from the peel to be moved in a plastic plate. At least five fruits were assayed by using a fruit hardness tester (Cat No. 9300, Tokyo-Japan) based on a method developed by Prasetia *et al*. (2018).

### Succinate Acid

Analysis of succinate acid was taken out in the testing laboratory of Saraswanti Indo Genetech, Bogor, West Java Province, Indonesia using a method proposed by Tagubase *et al*. (2016).

### Organoleptic Test

The 21 trained panellists were involved in evaluating the pulp colour, aroma, taste and texture. Panellists trained as organoleptic examiners aged 20 to 45 with an organoleptic analyst profession were split between 10 men and 11 women. Samples of durian fruit were presented as many as two pieces of durian fleshes carried out in an organoleptic test laboratory with a controlled temperature of around 22°C–24°C. The hedonic scale consists of 5-points Likert scale (5: like very much; 4: like; 3: moderate acceptance; 2: dislike; 1: dislike very much). Two types of samples were provided in four different periods each, in between 10 min of breaks and adding mineral water to neutralise the previous senses.

### Statistical Analysis

The experimental design used in this study was the Factorial Completely Randomised Design with three replications for each observations. The profiles of temperature–time relationships were processed by using Microsoft Excel 2010. While the periodic-values gained from the durian samples observations were statistically differentiated through ANOVA followed by Duncan Multiple Range Test (DMRT) for determining the further significant results using SPSS 20.0 and were expressed as mean ± S.E.

## RESULTS

### Relationship between the Time Process and the Core Freezing Temperature

**Figure 1:**
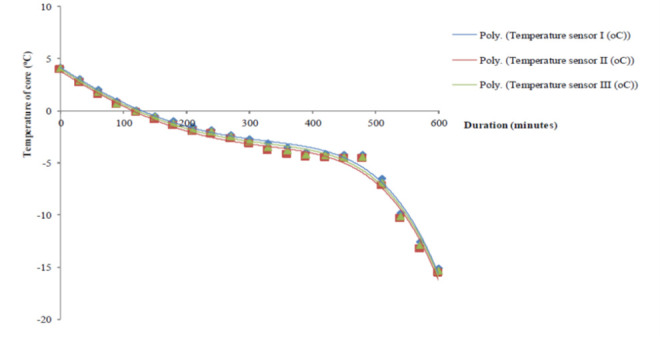
Core’s profiling temperature from the pre-cooling process, at 4°C, to the targeted freezing point, at −15°C.

A definite period was required for this process of declining temperature inside the durian fruit core until the targeted freezing temperature was achieved. A complete temperature oscillation during the treatment was described in Fig. 1. Initially, the trend was sharply declining. Then, it sharply decreased due to the defrosting cycle of time. Once the circulation of the cooled fresh air was retreated into a normal freezing, which was indicated by a steady pattern of lowering of the three temperature-probes, until the final temperature, as described in Fig. 1.

**Figure 2:**
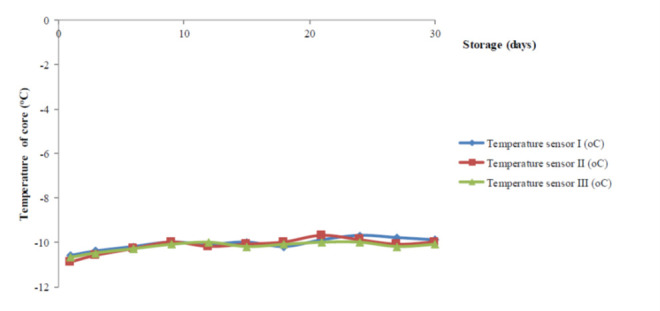
Profile of the core-temperature periodically observed during the freezing storage.

The entire temperature-loggers briefly showed a series of data having a close relationship with each other. Amongst the data obtained from this experiment; it is clear that the temperature sensor I have the best correlation (R^2^-value = 0.991) compared to the rest (Table 1). In the following step (freezing storage), two-targeted freezing temperatures was achieved while the three temperature-probes were in a consistent performance matching with the previous one (Fig. 2). Up to the sixth day of storage, a sharp decline in the temperature margins monitored on the three temperature sensors indicating a massive effort in reaching out to the targeted one (Table 2). Soon after, the targeted temperature has almost reached out, briefly described by the value shown by the entire final temperature profile in a steady pattern, supported by the standard errors less than 10% consistently observed until the end of storage.

### Impact of Freezing Storage on the Physical Characteristics of Durian’s Pulp

**Figure 3:**
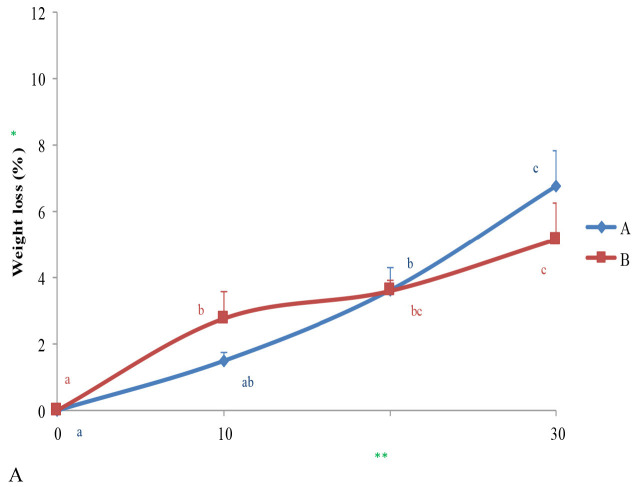

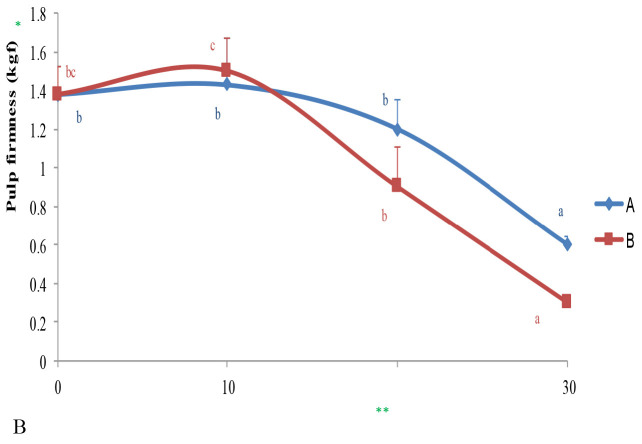

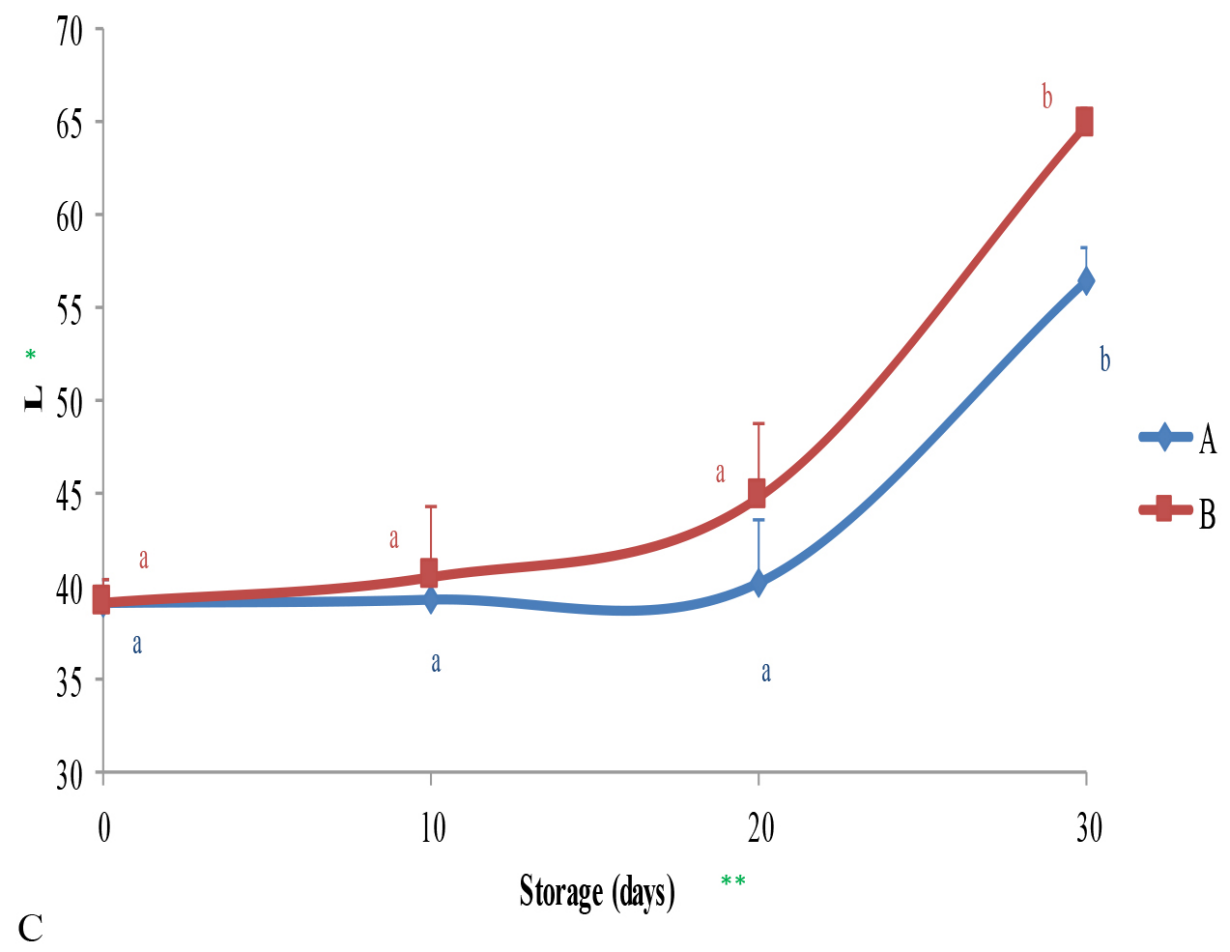

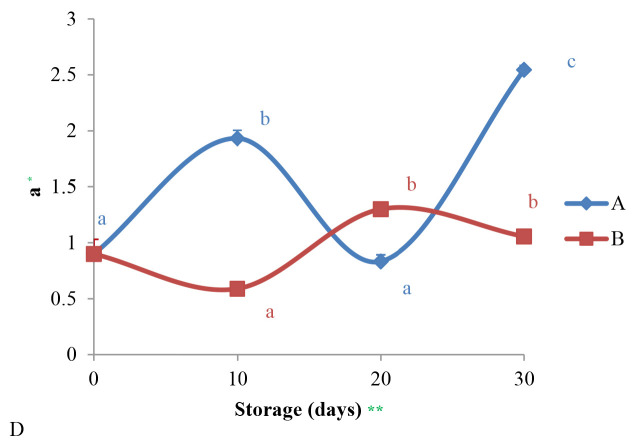

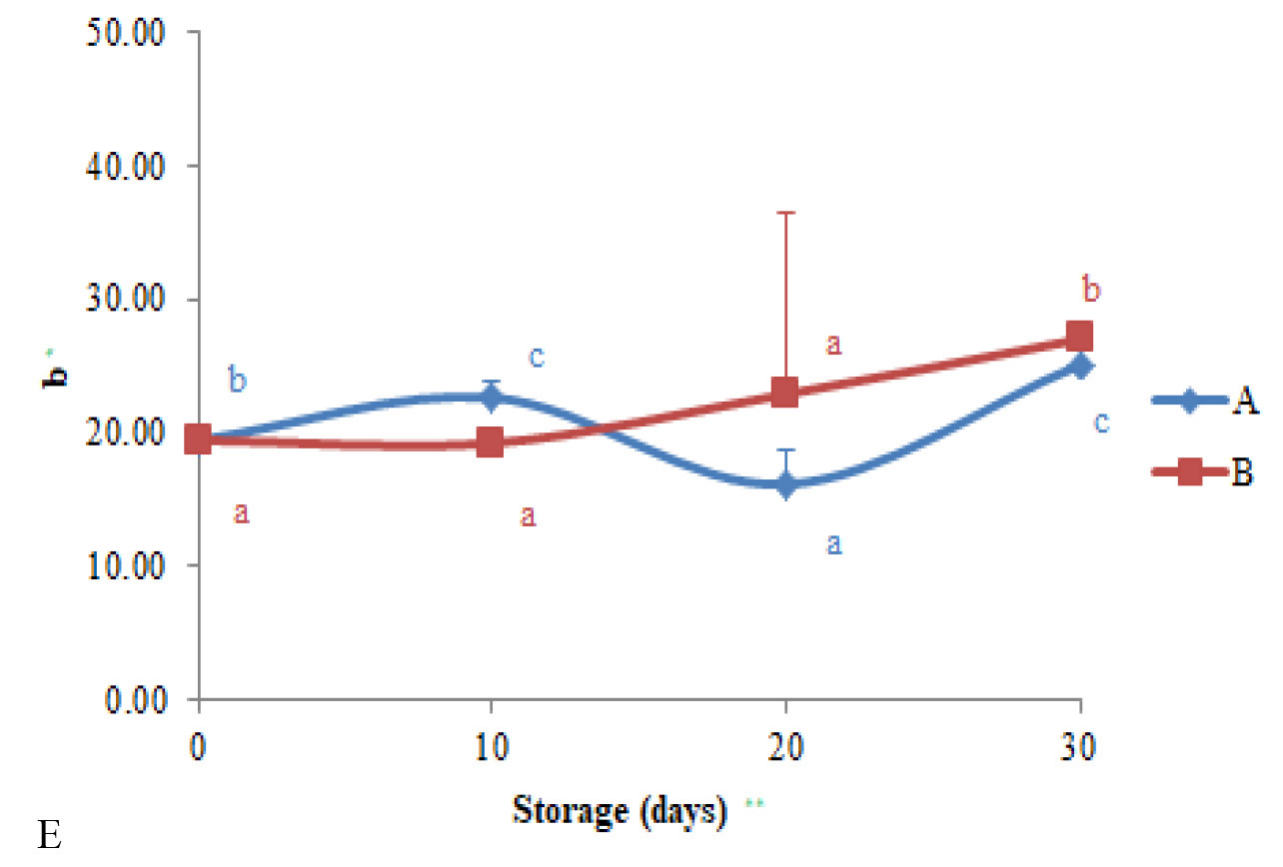

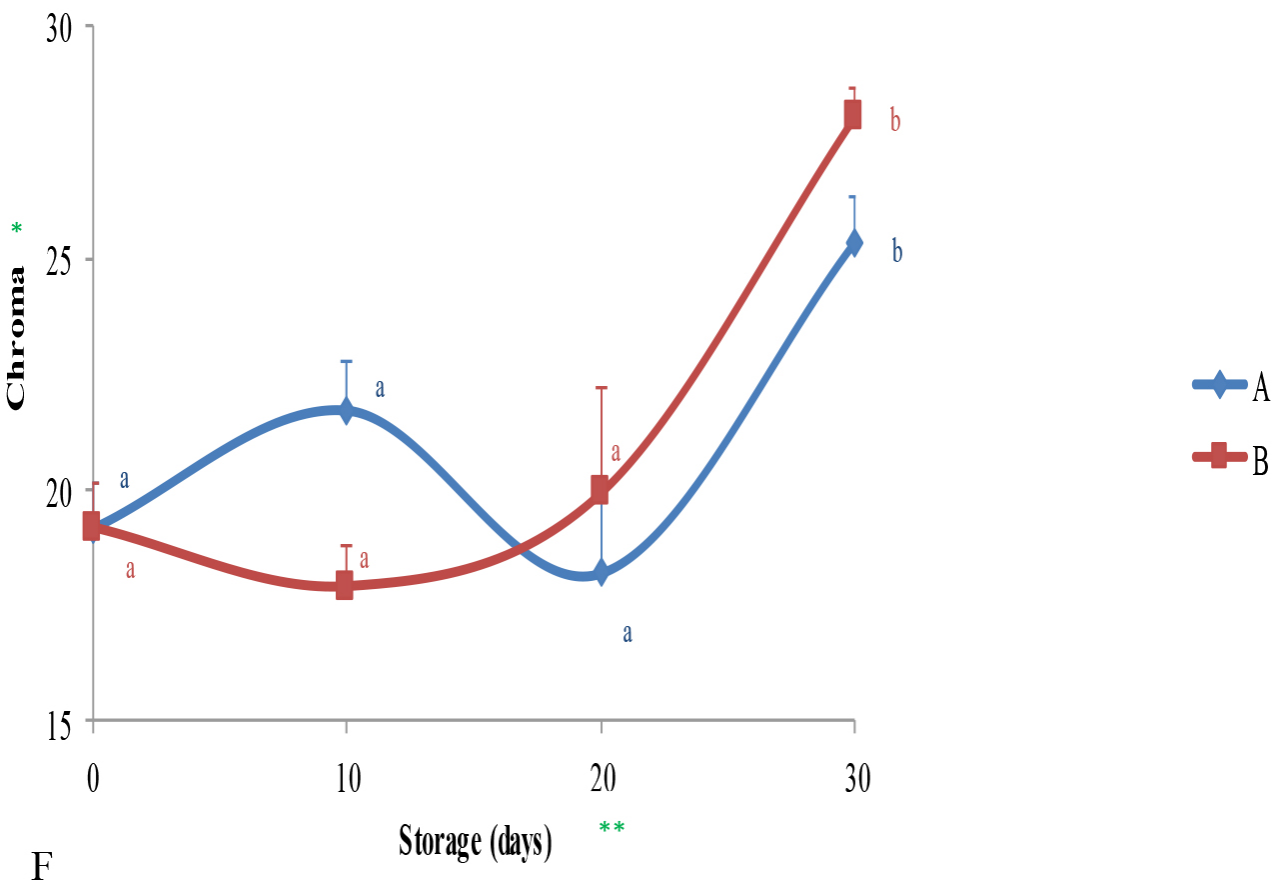

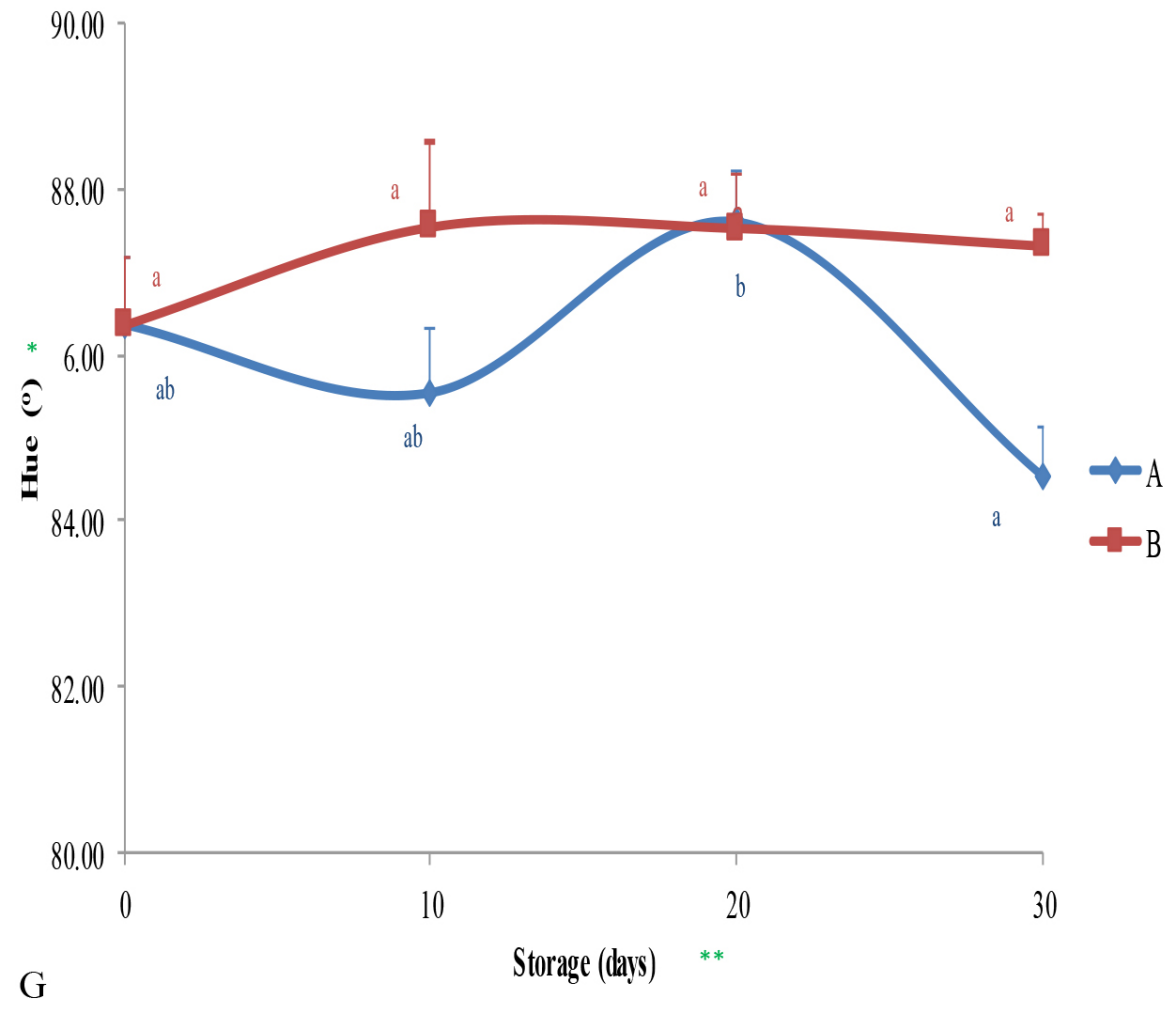
Changing in weights losses (A), fruit firmness (B), and colour characteristics (C–G) observed on whole durians var. *Langgang Kamang* in different level of treatment. *Results expressed as means ± SE; **Each of different lowercase letters indicating significant-results in between time storage-observations (*p* < 0.05) based on DMRT.

Physical characteristics such as the weight losses, flesh firmness and pulp colour was affected by the freeze-process and time. The effects of freezing on said characteristics was demonstrated in Fig. 3. Overall, the B-level of treatment moderately impact on controlling the inclination of weight losses until the end of the storage period (Fig. 3A).

This result was different compared to the previous findings, that is vice versa. For instance, the firmness profile of those fruits that was treated by both levels of treatments were steadily the same until the two-thirds period of freeze-storage. While the lowest values of flesh firmness observed at the end of storage were distinctly shown by the pulp have become softer and watery (Fig. 3B).

On the other hand, colour characteristics have regularly formed preferences, observed on fruits treated with the B-level. Except a-values were mostly low as well, raisings of lightness, b and chrome-values, and also hue angle have consistently taken on at each of the scheduled timelines. It means that fruits treated at B-level are distinctly more precise and consistent performances (Figs. 3C–3G) compared to the fruits treated at A-one. Moreover, entire trends of colour parameters have revealed that the colour characteristics were still acceptable, even though fruit’s treated with the A-level showed more fluctuated performance.

### Influence of Freezing Storage on Chemical Properties of Durian Fruits

**Figure 4:**
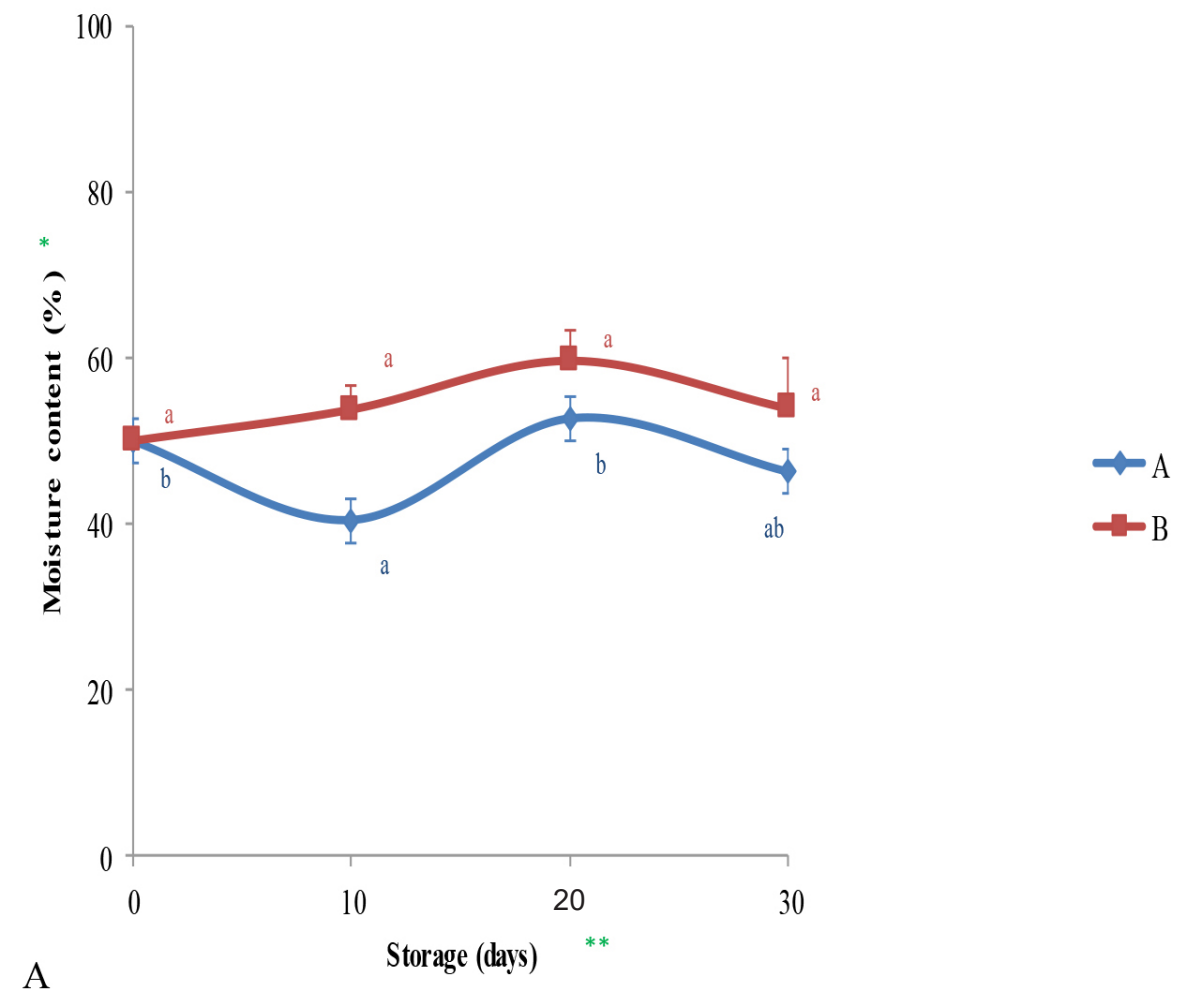

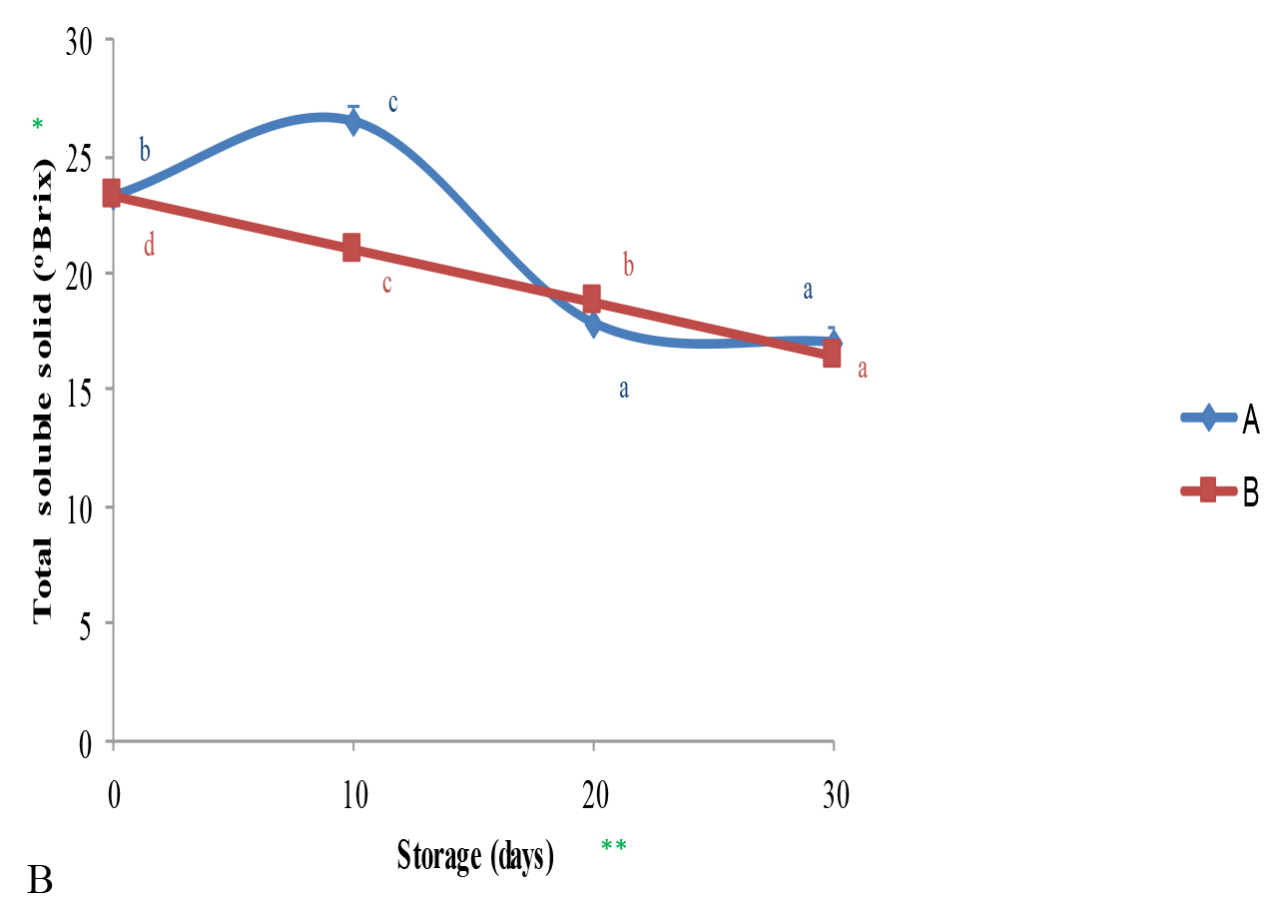

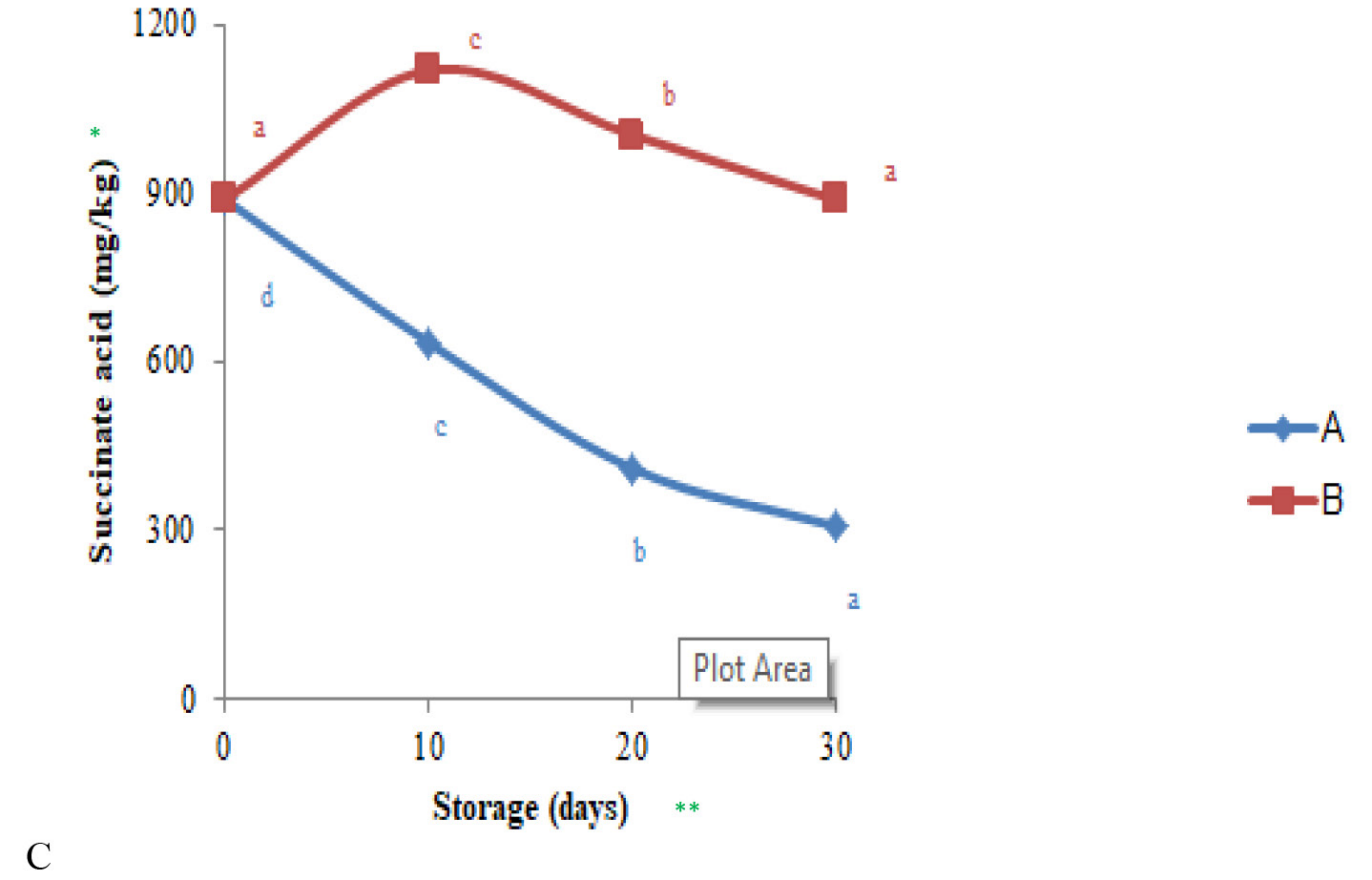
Altering in (A) moisture content, (B) total dissolved solid and (C) succinate acid monitored on the whole treated durians in disparate level of treatment. *Results expressed as means ± SE; **Each of different lowercase letters indicating significant-results in between time storage-observations (*p* < 0.05) based on DMRT.

Chemical properties such as moisture content, total of dissolved solids and succinate acid level were altered during freezing storage. Those alterations were based on the time-interval of storage as illustrated in Figs. 4A–4C. A less-fluctuating patterns of material humidity was fairly observed (Fig. 4A) on the fruits treated by the B-level. However, no significant differences were found on the material humidity between those two groups of the treated fruits.

Different from the previous findings, the trends of total soluble solids and organic acid were finally decreased. The correlation between the linear declining of total dissolved solid and the hyperbolic trend of succinate acid found on fruits treated by the B-level remain unclear. However, the fruit flavouring compound as well maintained this result was important in maintaining fruit flavouring agent traced by consumers, rather than the more oscillated result gained from the fruits treated with the A-level (Figs. 4B and 4C). This finding also confirmed why the peak maturity was not homogenously achieved yet at the end of storage-time.

### Preference Test to Freeze Durian Fruit

**Figure 5:**
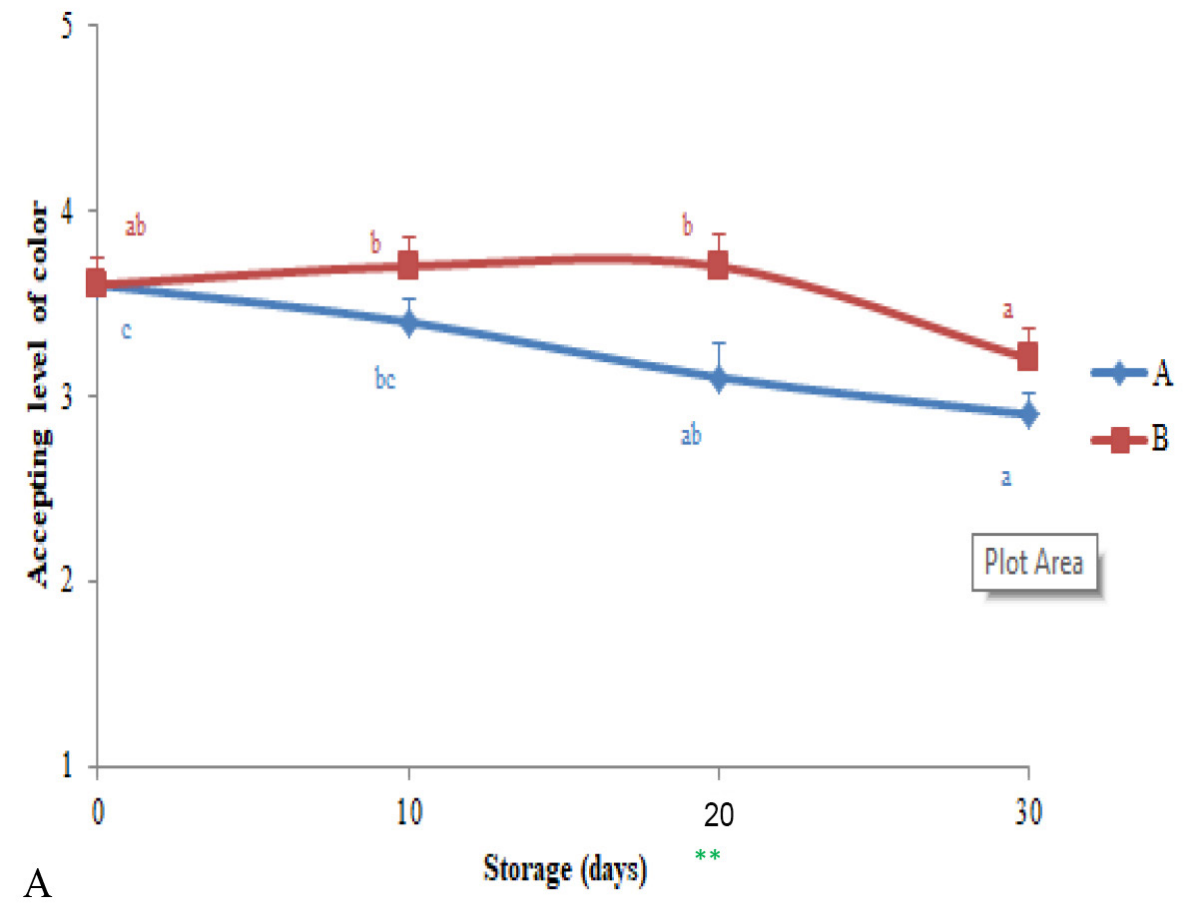

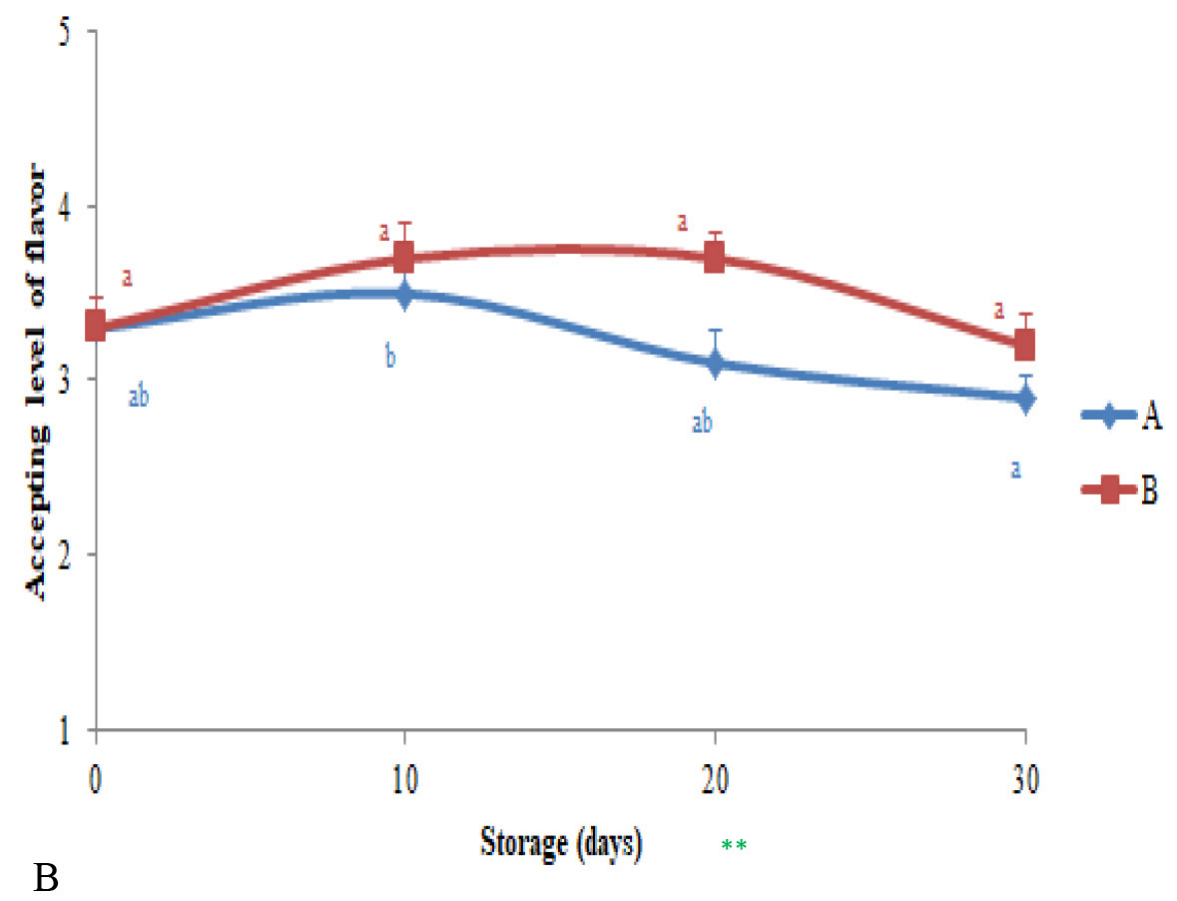

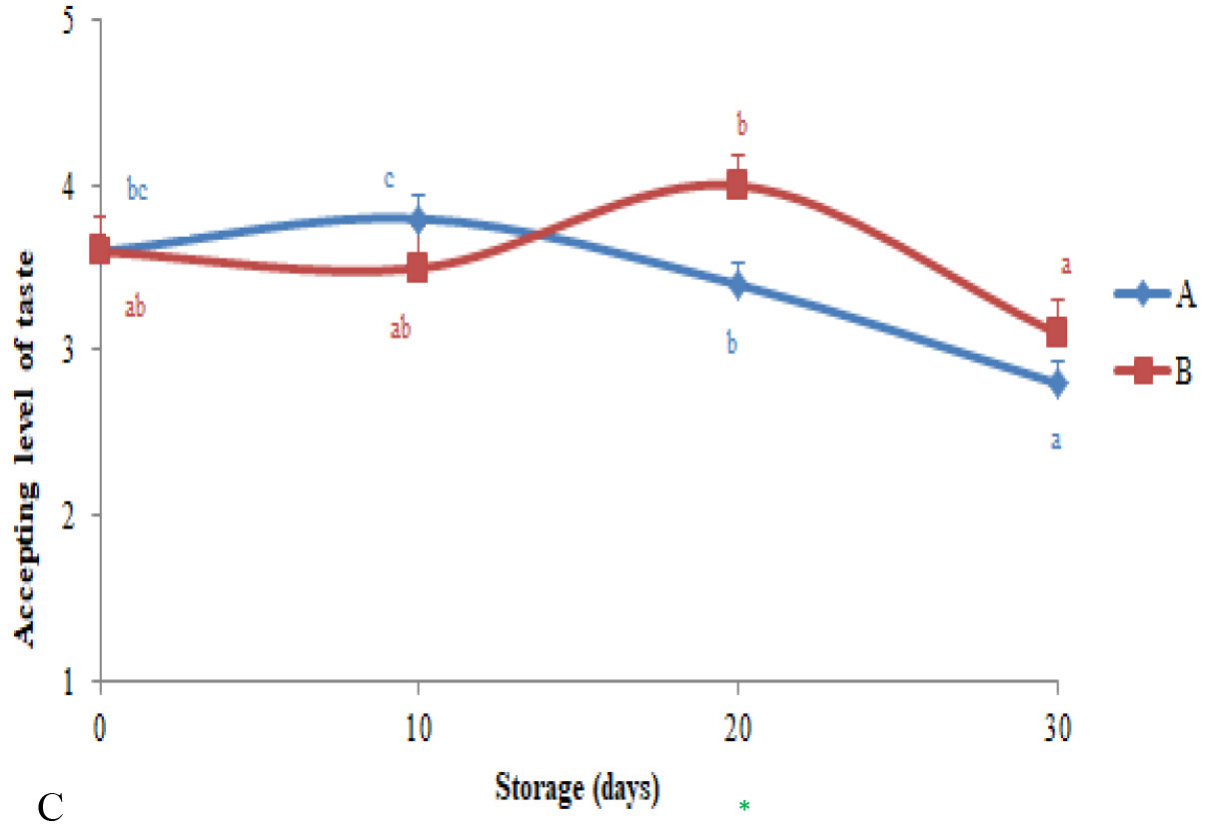

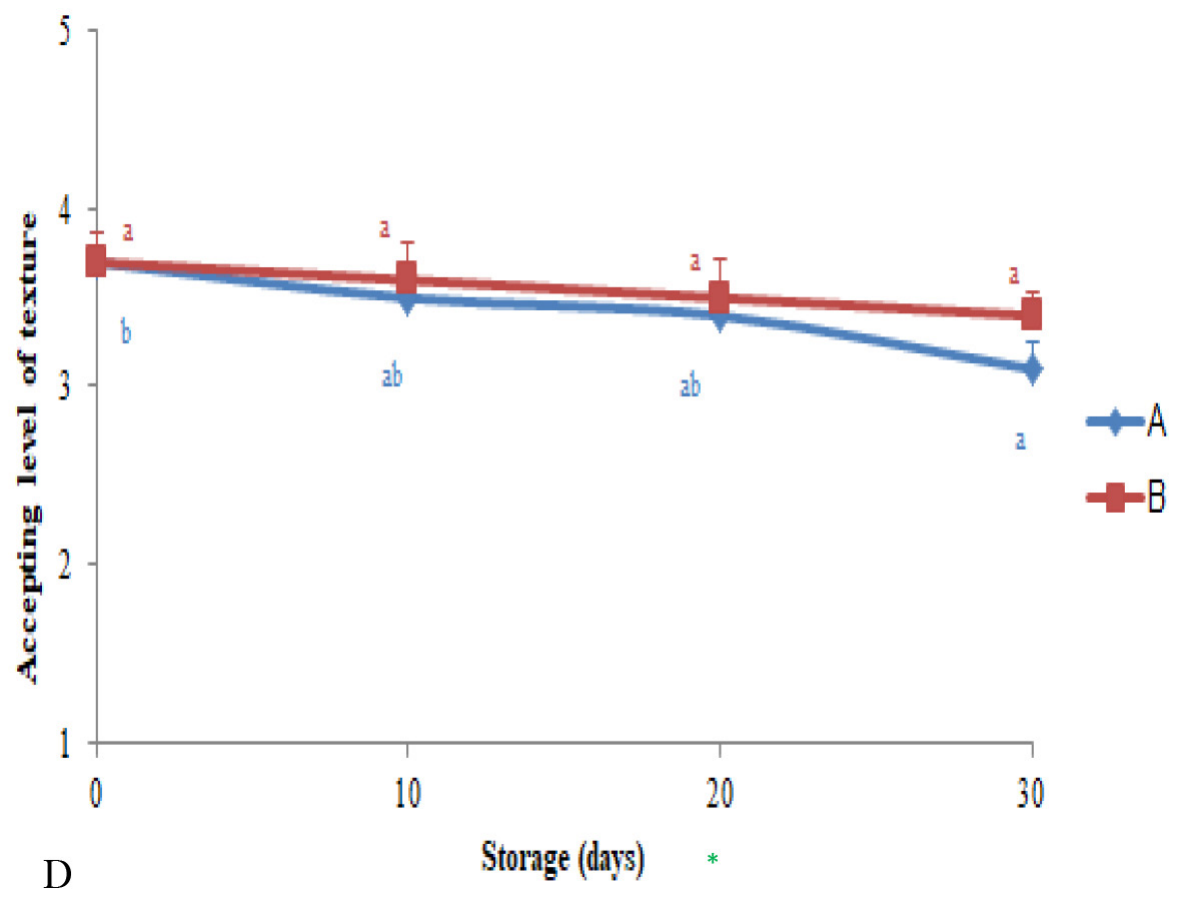
Respondents preferences to (A) colour, (B) flavour, (C) taste and (D) texture of fruits treated with the two-level of treatment. *Results expressed as means ± SE. **Each of different lowercase letters indicating significant-results in between time storage-observations (*p* < 0.05) based on DMRT.

No rejection was given by all panellists toward five parameters (colour, flavour, taste and texture) of freeze flesh durian treated by treatment A and B. However, the highest score was reached by fruit treated with B. A complete preference score based on time storage was shown in Figs. 5A–5D. There is an obvious fact faced on the tested fruits, ideally not in the peak ripeness. Therefore, the highest scores were not achieved until the end of storage time.

Predominantly, higher scores obtained in fruits treated with the B-level from panellists were affected by their perceptions on accepting physical appearances of the fruits. The brightness of colour, the sweetness of pulp, and the dominant fragrance of ripening fruits are the three-main indicator in making many more differences of respondent marks.

## DISCUSSION

Dry-freezing systems have several unique features when it comes to preserving horticultural commodities. Most of these systems have adequate capacities for covering a wide range of capabilities in suppressing many advanced reactions of biochemistry while maintaining essential nutrition, and also preventing the latent threat of microorganism contamination, making it suitable for extending the shelf life (Ciurzynska *et al*. 2014; Franceschinis *et al*. 2014; Harguindeguy & Fissore 2019; Santos & Silva 2008). Specifically, the heat transfer rate needs to be designed appropriately, particularly on minimising the excessive heat blockage. As one of the most common technical procedures, defrosting process has regularly been actuated for removal of this uncontrolled heat through compensating an extra accessible-ambient makeup in replacing the saturated air gradually to renormalize the operating temperature as the crucial feedback for the freezing process (Dong *et al*. 2011; Hossain 1995; Kim & Lee 2011; Salvadori *et al*. 1997).

When the final temperature has been achieved with a minimum fluctuation, this indicate that the targeted scheme is under control, as observed in our study. Consequently, the work-performance in between a series of specific thermo-sensors has indicated the homogenously distributed heat-transfer values occurred on the entire freezing process (Resende 2001). The result shown in Table 2 is also positively related to the previous research, mentioning a sharp decline of the heat transfer reaching almost zero-value has steadily correlated with the homogeneity of the final temperature finally reached out (Resende *et al*. 2002).

Furthermore, the selected thawing process was influenced by observing the fruit’s quality characteristics based on a time-series evaluation. As previously mentioned, the thawing process has positively affected inclining moisture content over time, brightening the pulp, and decreasing weight loss (Arancibia-Avila *et al*. 2008; Ikram *et al*. 2009; Tagubase *et al*. 2016). Our study has also confirmed with the previous studies mentioning softening of the pulp’s texture, declining of total suspended solids, and stabilising of organic flavour have been strongly related to high intensity of the low-temperature preservation (Burdon *et al*. 2017; Gwanpua *et al*. 2018; Ishiwu *et al*. 2014; Yang *et al*. 2013; Zhao *et al*. 2015). Our result also confirmed it was difficult, ideally for returning to the normal maturity process. This finding was closely associated with the abnormality of ethylene production drastically caused by a sharp decline of three enzymes activities, namely: ACS (1-aminocyclopropane-1-carboxylate synthase), ACO (1-aminocycloprapane oxidase), and ACC (1-aminocyclopropane-1-carboxylate) (Lara & Vendrell 2003), particularly carried on a range of extreme temperatures, including freeze environment (Maninang *et al*. 2011; Sirijariyawat & Charoenrein 2012).

Our study has also showed that a strong indication of delaying post-maturity due to the freezing storage as seen in a consistent pattern based on several previous studies (Burdon 2015; Burdon *et al*. 2016; Manning *et al*. 2016). Therefore, these products have become less sensitive towards several enzymatic activities when they are warmed (Chassagne-Berces *et al*. 2010) and therefore the dissolved solid’s profile has gradually declined due to an inclining of respiration rate observed on the late maturity (Harman 1987). In addition, a complex structure of durian together with post-freezing temperature alteration, has been suspected as the main factor in effecting the downtrends in dissolved solids (Alhamdan *et al*. 2016; Lara & Vendrell 2003). As regarded to be in a crucial role, thawing process has mostly affected in minimalising the loss of antioxidants as well as organic compound (Dantes *et al*. 2014). Our results confirmed that the two hyperbolic trends of succinic acid and moisture content, particularly observed on the fruits treated with B, were intimately related to the blocked enzymatic reaction. This was also true for the fully inhibited ripe process (Maninang *et al*. 2011; Moya-Leon *et al*. 2006; Rizzolo *et al*. 2005). It is a strong indication in evaluating the fruits as no perfectly ripe ones concerning some particular treatments for achieving the targeted criteria.

Furthermore, sensory characteristics mark a specific preference. This has been an area of interest, mostly for differentiating the interests of customers. To address this issue, most Asians prefer fully ripened fruits (Nanthachai *et al*. 1994; Maninang *et al*. 2011). Some prefer a more juicy and less sweet taste. The odours of sulphur and esters containing substances subjectively traced, aligning to the full-ripe fruits indication, were regulated by a thermodynamic equilibrium (Defilippi *et al*. 2005), ideally hard to be achieved for this study. Whereas the flat-pattern of texture gained in this research, was strongly correlated to the stability of flesh water holding capacity, mainly important on determining food sensory preferences (Sriyook *et al*. 1994; Tagubase *et al*. 2016).

## CONCLUSION

The freezing process applied to the whole durian fruit (−15°C for 20 min at the core followed by −10°C for 30 days) exhibited the maximum impact on conserving the stability of physics-chemical properties. The results gained from the organoleptic evaluation proved that the overall fruit characteristics were still acceptable by the panellists. Therefore, freezing could be considered as a prospective model, particularly for freezing preservation. Fruit’s deteriorating could be overcome by an advance time of treatment (30 days) without any significant decrease on the fruit’s quality. However, further research is still required to verify this recent inventory.

## Figures and Tables

**Table 1:** Summary of the proposed models describing the relations between the measured core’s temperature (y) and the required time (x) on achieving the targeted freeze point, at −15°C.

Number of probes	Polynomial equation	Correlation (R^2^-values)
I	y = −8 10^−13^ x^5^ + 5 10^−10^ x^4^ − 10^−7^ x^3^ + 7 10^−5^ x^2^ − 0.04 x + 4.18	0.991
II	y = −6 10^−13^ x^5^ + 3 10^−10^ x^4^ +10^−8^ x^3^ + 4 10^−5^ x^2^ − 0.04 x + 3.81	0.989
III	y = −7 10^−13^ x^5^ + 4 10^−10^ x^4^ −7 10^−8^ x^3^ + 6 10^−5^ x^2^ − 0.04 x + 4.03	0.990

**Table 2:** The absolute margins between the recorded temperature (T_r_), and the targeted one (T) at −10°C, and the average margin temperature equipped with the standard errors (SE) observed during the freezing storage period.

Storage (days)	|T_r_ – T |_I_	|T_r_ – T |_II_	|T_r_ – T |_III_	Δ|T_r_ – T |_avg_ ± SE
0	0.6	0.9	0.7	0.73 ± 0.023
3	0.4	0.6	0.5	0.50 ± 0.010
6	0.2	0.3	0.3	0.27 ± 0.003
9	0	0	0.1	0.03 ± 0.003
12	0.1	0.2	0	0.10 ± 0.010
15	0	0.1	0.2	0.10 ± 0.010
18	0.2	0	0.1	0.10 ± 0.010
21	0.1	0.3	0	0.13 ± 0.023
24	0.3	0.1	0	0.13 ± 0.023
27	0.2	0.1	0.2	0.17 ± 0.003
30	0.1	0	0.1	0.07 ± 0.003
